# Versatile Roles of Aquaporins in Plant Growth and Development

**DOI:** 10.3390/ijms21249485

**Published:** 2020-12-13

**Authors:** Yan Wang, Zhijie Zhao, Fang Liu, Lirong Sun, Fushun Hao

**Affiliations:** 1 State Key Laboratory of Cotton Biology, Henan Key Laboratory of Plant Stress Biology, School of Life Sciences, Henan University, Kaifeng 475004, China; wy875643001wy@163.com (Y.W.); zhaozhijie2021@126.com (Z.Z.); 2 State Key Laboratory of Cotton Biology, Institute of Cotton Research of CAAS, Anyang 455004, China; liufcri@163.com

**Keywords:** aquaporins, seed germination, root growth, shoot elongation, leaf development, reproductive development, gene expression

## Abstract

Aquaporins (AQPs) are universal membrane integrated water channel proteins that selectively and reversibly facilitate the movement of water, gases, metalloids, and other small neutral solutes across cellular membranes in living organisms. Compared with other organisms, plants have the largest number of AQP members with diverse characteristics, subcellular localizations and substrate permeabilities. AQPs play important roles in plant water relations, cell turgor pressure maintenance, the hydraulic regulation of roots and leaves, and in leaf transpiration, root water uptake, and plant responses to multiple biotic and abiotic stresses. They are also required for plant growth and development. In this review, we comprehensively summarize the expression and roles of diverse AQPs in the growth and development of various vegetative and reproductive organs in plants. The functions of AQPs in the intracellular translocation of hydrogen peroxide are also discussed.

## 1. Introduction

Aquaporins (AQPs) are ubiquitous membrane channel proteins that selectively and reversibly facilitate water movement across plasmalemma and organelle membranes in plants and other organisms [1,2]. In growing plants, water is constantly absorbed by roots, flows axially through xylem vessels and moves radially through apoplastic, symplastic and transcellular pathways to leaves, and evaporates through stomata. In the transcellular pathway, water traverses cellular membranes either by simple diffusion or more frequently by AQP-formed pores [1,2]. The rapid movement of water through the biomembrane is essential for the maintenance of cellular water homeostasis as well as for the accomplishment of various metabolic activities under many circumstances, for example during cell elongation, root water absorption, leaf movement, stomatal opening and closure, flowering and fertilization. Therefore, AQPs are greatly important for plant growth, development and survival [1,2,3,4].

AQPs transport not only water and H_2_O_2_, but also other neutral molecules with important physiological significance such as carbon dioxide (CO_2_), glycerol, ammonium, urea, and metalloids like boric acid, silicic acid and arsenic acid [2]. Some AQPs may also have the ability to conduct monovalent cations [5,6]. AQPs have been found to fulfill functions in responses to a variety of stresses like drought, osmotic stress, high salinity, cold, anoxia, nutrient unavailability and pathogen attack; in growth and development including seed germination, root growth, stem (shoot) elongation, leaf expansion, and reproductive organ development; and in the signal transduction of multiple hormones such as auxin, gibberellins (GAs), ethylene and abscisic acid (ABA) in plants [1,2,3,7].

In recent years, many review papers covering the structure, classification, localization, substrate specificity, trafficking, functions and regulation of AQPs have been published [2,3,7,8,9,10]. However, the roles of AQPs in the growth and development of diverse plant organs have not been comprehensively reviewed. Here, we mainly discuss the expression and functions of various AQPs during plant growth and development.

## 2. Classification, Characteristics and Regulation of Plant AQPs

AQPs belong to the membrane integrated major intrinsic superfamily proteins (MIPs) with small molecule weights (about 26 to 34 kDa), and are present in nearly all living organisms. Besides AQPs, MIPs contain two subfamily proteins: glycerol facilitators (GLPs/GlpFs) and aquaglyceroporins (GLAs). GLPs transfer glycerol and neutral molecules, and GLAs transport water, glycerol and other small solutes. All MIPs are comprised of six membrane-spanning alpha helices, five inter-helical loops, an Ala-Glu-Phe (AEF or AEFXXT) motif, two highly conserved Asp-Pro-Ala (NPA) motifs and an aromatic/arginine (ar/R) region. Both the NPA motifs and ar/R selectivity filter are important for determining the substrate selectivity of AQPs [8,11].

In cellular membranes, AQPs form homo- or hetero-tetramer pores, and each of the four subunits can generate a water channel pore for permeability of substrate molecules [2,3,8]. Based on their intracellular locations and sequence similarities, AQPs are categorized into five major subfamilies in higher plants: the plasma membrane intrinsic proteins (PIPs), tonoplast intrinsic proteins (TIPs), nodulin 26-like intrinsic proteins (NIPs), small basic intrinsic proteins (SIPs) and uncategorized intrinsic proteins (XIPs) [12]. Among these, PIPs and TIPs are predominant, and mainly mediate water flow across cells and subcellular compartments in plants. PIPs can be further divided into PIP1 and PIP2 subgroups. Each subgroup contains different isoforms named PIP1;1, and PIP1;2, etc. Similar to PIPs, TIPs are further classified into TIP1 (formerly called γ-TIP, similarly hereinafter), TIP2 (δ-TIP), TIP3 (α-TIP or β-TIP), TIP4 (ε-TIP) and TIP5 (ξ-TIP) subtypes, and the isoforms within these subtypes are designated as TIP1;1, TIP1;2, and so on. NIPs are also divided into NIPIs, NIPIIs and NIPIIIs based on their channel pore structures. NIPIs transport water, glycerol and lactic acid, while both NIPIIs and NIPIIIs are mainly permeable to metalloids such as silicic acid, boric acid, arsenic acid, selenite and germanic acid [13].

At present, AQP family members have been identified or investigated in more than 100 plant species [14,15]. Compared with animals and other organisms, plants contain the largest number of AQP homologs. Moreover, AQPs in plants are highly diverse in species, subcellular localizations, spatiotemporal expression patterns, solute permeability and functions [1,2,3,7]. The functional efficiency of an AQP is determined by its amounts and activity (opened or closed states, or gating) in the membranes, as well as its specific permeability for substrates. The abundance of an AQP is regulated by transcriptional or post-transcriptional processes. The activity of an AQP is controlled by posttranslational modifications (for example, phosphorylation and methylation), pH, Ca^2+^, interactions between one AQP with other AQPs or proteins, while the substrate specificity of an AQP is governed by its architecture [2,3,16,17,18,19]. The localizations, substrate permeability, properties and regulations of AQPs in plants have been widely reviewed in recent years [2,20,21].

## 3. Roles of AQPs in Vegetative Growth

### 3.1. Seed Germination

Seed germination is fundamental for seedling establishment. It commences with imbibition, followed by rehydration of the endosperm and embryo, cell vacuolation (large vacuole biosynthesis), metabolic activation, reserve mobilization, the elongation of the embryonic axis and radicle emergence. Most of these processes are inseparable from AQP-facilitated water uptake and transport in seeds [22,23,24,25].

Expression analysis revealed that many TIP1, TIP2 and PIP genes are strongly expressed or noticeably upregulated in mRNA and/or protein levels, whereas TIP3s are significantly downregulated during seed germination in many plants, highlighting their possible roles in germination [23]. The upregulated genes mainly include *AtTIP1;1*, *AtTIP1;2*, *AtTIP2;1*, *AtTIP2;2*, *AtPIP1;1*, *AtPIP1;2*, *AtPIP1;3*, *AtPIP1;4*, *AtPIP2;1*, *AtPIP2;2* and *AtPIP2;7 in Arabidopsis* [25,26,27,28]; *OsTIP1;1*, *OsTIP1;2*, *OsPIP1;1*, *OsPIP1;2*, *OsPIP1;3*, *OsPIP2;1*, *OsPIP2;4*, *OsPIP2;5*, *OsPIP2;7* and *OsPIP2;8 in rice (Oryza sativa*) [29,30]; *PsTIP1;1* and *PsPIP1;1 in pea (Pisum sativum*) [31]; *Bnγ-TIP2*, *BnPIP1* and *BnPIP1;4 in oilseed rape (Brassica napus*) [32,33]; and *VfTIP1;1*, *VfTIP2;1*, *VfTIP2;2* and *VfPIP2;1 in broad bean (Vicia faba* var. *minor*) [34]. In contrast, transcripts and/or proteins of *AtTIP3;1* and *AtTIP3;2* of *Arabidopsis* [26,28], *OsTIP3;1* and *OsTIP3;2* of rice [30], and *VfTIP3;1* and *VfTIP3;2* of broad bean [34] dramatically decrease or vanish during seed germination (Figure S1).

**Figure 1:**
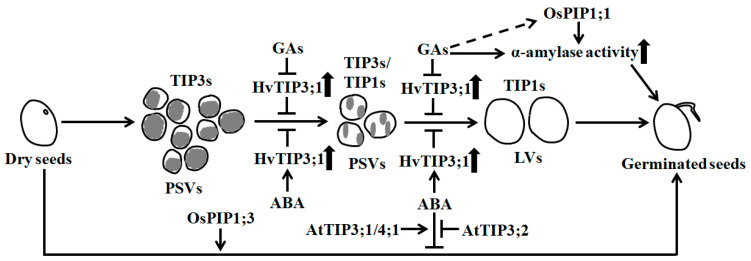
Aquaporins are required for the germination of orthodox seeds through affecting vacuolation and α-amylase activity. During seed germination, vacuolation occurs. TIP3s are enriched in small protein storage vacuoles (PSVs), whereas TIP1s accumulate in large vacuoles (LV). TIP3s negatively affect the formation of LVs. Gibberellins (GAs) activate α-amylase activity and promote the conversion of PSVs to LVs by inhibiting *HvTIP3;1* expression, but abscisic acid (ABA) has the opposite effect during barley seed germination [38]. AtTIP3;1 and AtTIP4;1 have positive effects, whereas AtTIP3;2 has negative effects on ABA-inhibited seed germination [39]. Rice OsPIP1;1 has a beneficial role in seed germination by inducing α-amylase activity, and OsPIP1;3 plays a positive role during seed germination [35]. Long solid arrows show the transition of seed germination, and short and thick up arrows indicate increases in the abundance of HvTIP3;1 and the activities of α-amylase. Short and thin solid arrows reveal positive regulation, and bars represent negative regulation. The dotted arrow represents unidentified regulation and the gray in PSVs shows the storage nutrients.

Genetic evidence further reveals that AQPs are required for seed germination. For instance, in rice, knock-down of *OsPIP1;3* leads to a clear reduction in seed germination rate under normal conditions [29]. Moderate or low overexpression of *OsPIP1;1* also significantly enhances the seed germination rate and the activity of α-amylase [35] (Figure 1) (Table 1).

In germinating seeds, some TIPs like TIP1s have been shown to function in vacuolar biosynthesis and in facilitating water flow into vacuoles, causing the mobilization of reserve substances, the establishment and sustainment of cell turgor pressure and the promotion of embryo cell elongation. Many PIPs such as PIP1s and PIP2s are found to act in water exchange between extracellular and cytoplasmic compartments, and are a requisite for water balance maintenance in the cytoplasm [22,23,36]. In dry orthodox seeds of higher plants, large amounts of nutrients like storage proteins have accumulated in many small protein storage vacuoles (PSVs) of cells. During germination, accompanied by the rapid flow of water into seeds through AQPs, the PSVs are fused and converted into large central lytic vacuoles (LVs), leading to the activation of multiple hydrolytic enzymes and a significant increase in cellular turgor pressure [23,37] (Figure 1). Thus, cell elongation is favored. TIP3s, the seed-specific expressed proteins, have been proven to be abundant in PSVs, while TIP1s are highly accumulated in LVs. During the formation of LVs in seed germination, the contents of TIP1s pronouncedly increase, while those of TIP3s drastically decrease or disappear [26,30,34]. The abundance of TIP1s are positively correlated to the formation of LVs, but those of TIP3s are negatively correlated [23,34] (Figure 1). Nevertheless, TIP3s are also found to favor optimal water uptake during the early stage of seed germination in *Vicia faba* [37].

In barley (*Hordeum vulgare*), the transformation of small PSVs into LVs in the aleurone cells of seed endosperm is modulated by gibberellins (GAs) and abscisic acid (ABA), two controllers of seed germination [60]. GAs promote the conversion of PSVs to LVs during seed germination, but ABA prevents it. GAs also specifically inhibit the expression of *HvTIP3;1* and *HvTIP1;2*, while ABA prominently enhances the transcription of *HvTIP3;1*. The increase and deletion of *HvTIP3;1* transcripts individually result in the delay of GA-induced vacuolation (coalescence of PSVs) and the stimulation of vacuolation, thereby inhibiting and promoting seed germination, respectively [38] (Figure 1). These results demonstrate that the transcriptional changes in *HvTIP3;1*, which are tightly controlled by GAs and ABA, are important for vacuolation and seed germination. However, the mechanism for HvTIP3;1 functioning in vacuolation is currently unknown. In *Arabidopsis*, genetic evidence shows that AtTIP3;1 and AtTIP4;1 are positive regulators of the ABA response, whereas AtTIP3;2 is a negative regulator of the ABA response during seed germination [39] (Figure 1). Thus, AtTIP3;1/4;1 and AtTIP3;2 antagonistically affect ABA-inhibited seed germination.

AQPs may also serve roles in the germination of recalcitrant seeds. In horse chestnut, AhTIP2, AhTIP3;1, AhPIP1 and AhPIP2 are enriched during seed germination. Active vacuoles that are maintained in the embryonic axis cells of hypocotyls and radicles after seed shedding initially enlarge, followed by vacuolation during seed germination. Moreover, cell vacuolation is accompanied by the activation of vacuolar acid invertase (breaking down the storage compound sucrose). After growth initiation, vacuole enlargement is facilitated by an AQP-mediated increase in water inflow [61].

### 3.2. Root Growth

Primary roots come from radicles in seeds. After seed germination, the growth of radicles is accompanied by the generation of LVs through the fusion of provacuoles in orthodox seed plants, or through the enlargement of preserved vacuoles in recalcitrant seed plants [23,61]. The formation of LVs is a prerequisite for cell elongation in that LVs act as osmotic compartments and produce turgor pressure to stimulate cell expansion. Evidence reveals that TIPs and PIPs are important for the syntheses of provacuoles and LVs through transferring water into vacuoles and cytoplasms during post-germinative root growth [22,23]. Consistently, *VfTIP2;1* and *VfTIP2;2* are highly expressed during post-germinative root growth in broad bean. Furthermore, the expression patterns of the two *AQPs* are in parallel with the formation of vacuoles from provacuoles in the meristems [34].

Apart from being expressed in radicles, PIPs and TIPs are enriched in the growing regions of roots or the root tip, where more growing cells and tissues exist [62,63,64]. In maize (*Zea mays*), *ZmPIP1;2*, *ZmPIP2;4* and *ZmTIP1;1* are highly expressed in the root growing zone [65]. Transcripts of *ZmPIP1;1*, *ZmPIP1;5*, *ZmPIP2;1*, *ZmPIP2;5* and *ZmPIP2;6* are abundant in the root tip, and the expression of most of the genes is developmentally regulated [66]. In barley, *HvPIP1;2*, *HvPIP2;2*, *HvPIP2;5*, *HvTIP1;1* and *HvTIP2;3* are strongly expressed in the growing tissue of roots [62]. *VvPIP1;1*, *VvPIP1;2*/*1;4*, *VvPIP1;3*/*1;5*, *VvPIP2;1*, *VvPIP2;2*, *VvPIP2;3* and *VvPIP2;4* are also dominantly expressed in the root tip of grapevine (*Vitis vinifera*), and the average transcript abundances of *VvPIP1;1*, *VvPIP1;2*/*1;4* and *VvPIP1;3*/*1;5* in the meristematic zone are 4–100 fold higher than in older root zones [63,67,68]. Moreover, the expression patterns of these *VvPIPs* are very similar to their homologs in *Arabidopsis,* including *AtPIP1;1*; *AtPIP1;2*, *AtPIP1;3*, *AtPIP2;1*; *AtPIP2;3* and *AtPIP2;6* [63,69] (Figure S1). The presence of high amounts of PIPs and TIPs in growing tissues might imply that these AQPs play important roles during root cell growth.

**Figure 2:**
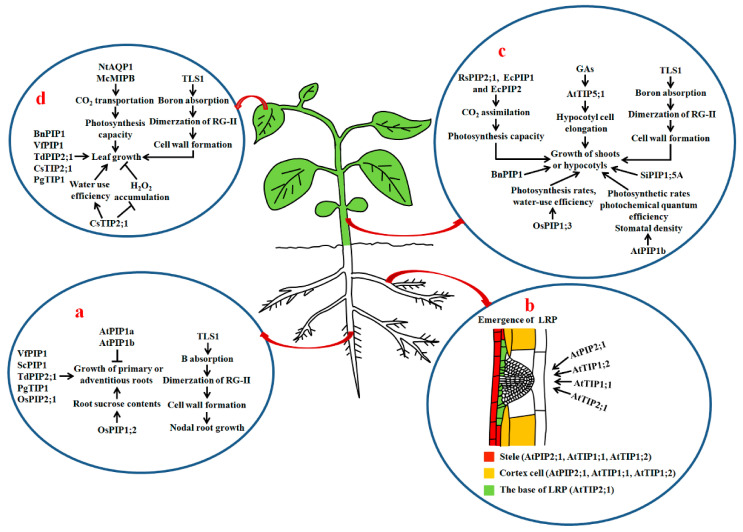
The roles of AQPs in the growth of roots, shoots, hypocotyls and leaves in plants. (**a**) Roles of AQPs in growth of primary roots, adventitious roots and nodal roots; (**b**) effects of PIPs and TIPs on lateral root formation. The expression or localization of AtPIP2;1, AtTIP1;1, AtTIP1;2 and AtTIP2;1 is shown in color; (**c**) roles of AQPs in shoot growth and hypocotyl elongation; (**d**) actions of AQPs in leaf growth. Thick up arrows represent the increase in *L*_pr_, the thin arrows show positive regulation, and bars show negative regulation. GAs: gibberellins; *L*_pr_: root hydraulic conductivity; LRP: lateral root primordium; RG-II: rhamnogalacturonan II; TLS1: maize tassel-less1.

The attributes of PIPs and TIPs affecting root growth were further confirmed by transgenic experiments. In *Arabidopsis*, antisense plants with decreased expression of *AtPIP1a* and *AtPIP1b* have remarkably abundant roots compared to the control plants under normal condition [40], indicating that the two PIP1s have negative effects on root development, or the increased roots compensate for reduced cellular water permeability. Conversely, the overexpression of *Vicia faba VfPIP1* in *Arabidopsis* significantly promotes the growth of primary and lateral roots (LRs) [41]. Transgenic *Arabidopsis* plants overexpressing Jojoba (*Simmondsia chinensis*) *ScPIP1* also exhibit longer roots and better growth status than the wild type (WT) plants under normal growth condition [42]. The reason for this may be that both VfPIP1 and ScPIP1 are homologous proteins in the plasma membrane, likely having similar structures and functions in root growth. Besides PIPs, transgenic durum wheat (*Triticum turgidum* subsp. *durum*) cv. Maali expressing wheat *TdPIP2;1*, and transgenic *Arabidopsis* plants overexpressing of *Panax ginseng PgTIP1,* display increased root growth [43,44] (Figure 2a).

Péret et al. demonstrated that auxin stimulates LR development through regulating the spatial and temporal distribution of AQP-dependent water transport in root tissues of *Arabidopsis*. Auxin decreases *AtPIP2;1* expression in cortical cells, but increases *AtPIP2;8* expression at the base of the lateral root primordium (LRP) and underlying stele when LRPs emerge. Both overexpression and disruption of *AtPIP2;1* causes defective LRs to emerge, suggesting that tight and spatiotemporal control of the AQP-mediated water flow in root tissues by auxin is necessitated for LR branching [45]. Recently, Reinhardt et al. reported that loss-of-function mutations of *AtTIP1;1*, *AtTIP1;2* and *AtTIP2;1* markedly inhibit the emergence of LRPs and decrease the number of LRs in *Arabidopsis*. They also found that the distributions and contents of the AtTIPs in the tonoplast of LRP cells modulate LR development. During LR formation, *AtTIP2;1* is initially expressed in some defined cells at the base, then actively expressed at the flanks in LRPs. Moreover, disruptions of *AtTIP1;1* and *AtTIP1;2* in LRPs clearly delay the emergence of LRs [46] (Figure 2b) (Table 1). Accordingly, both the PIPs and TIPs act in the establishment of LRP patterning and the formation of LRs via finely controlling water flow at the sites of LR emergence in plants.

In rice, the overexpression of *OsPIP1;2* leads to substantial enhancements of leaf CO_2_ conductance, photosynthetic performance and sucrose transport activity in the shoot phloem, and of root sucrose contents and root length [47]. Noteworthily, phloem sucrose transport from shoot to root exerts essential effects on root growth [70]. Moreover, sucrose influences root development as a signaling molecule and as an energy source [71]. Therefore, AQPs can play a role in root growth through facilitating leaf CO_2_ diffusion and phloem sucrose transport in plants (Figure 2a) (Table 1).

Root growth is mostly determined by cell division, elongation and differentiation in the root tip of plants. These cellular behaviors, especially cell elongation, are largely dependent upon the proper hydraulic conductivity of roots (*L*_pr_) and root cells (*L*_prc_), which are regulated by the functions of AQPs [2,3,72]. Pharmacological data reveal that AQPs contribute to the *L*_pr_ up to 64% in *Arabidopsis* [73], 70–80% in wheat (*Triticum aestivum*) [74], 60–70% in maize [65,75], 57% in tomato (*Solanum lycopersicum*) [76], and >90% in barley [62]. The mutation of *AtPIP2;2* in *Arabidopsis* has been shown to decrease *L*_prc_ by about 25–30% [77]. Null mutant *atpip1;2* displays a 20–30% reduction in *L*_pr_ [78]. Likewise, the inhibition of pea *PsPIP2;1* expression by the VIGS method causes a significant blockage of water transport, and about a 29% and 20% reduction of *L*_pr_ and *L*_prc_, respectively [79].

In maize, the *L*_prc_ values in two transgenic lines overexpressing *ZmPIP2;5* increase by 67% and 69%, whereas *L*_prc_ values in a *Zmpip2;5* knockout line decrease by 63% compared with the control [80]. The knock-down of *OsPIP2;1* by RNAi in rice also leads to a decline in *L*_pr_ by approximately four fold. Compared to WT, the RNAi plants have markedly lowered root total length, surface area, root volume and fewer root tips [48] (Figure 2a) (Table 1).

Consistent with the roles of AQPs in the adjustment of *L*_pr_ and *L*_prc_, and of root growth, *L*_pr_ in the meristematic and elongation zones is about 10 times greater than that in the secondary growth zone in grapevine fine roots. The inhibitory effects of the AQP blocker H_2_O_2_ on *L*_pr_ in the meristematic and elongation zones are far greater than those in the secondary growth zone [63]. Similarly, the *L*_prc_ in the growth zone is about 100 times higher than in the mature root region during gravitropic bending in pea roots [81].

Additionally, AQPs can exert effects on root growth by impacting the effective absorption of water and some nutrients from soil, and the subsequent distributions of these substances in plants [1,2,3,77,82]. It is known that the driving forces for water uptake and transport come from the potential differences of water along not only xylem vessels (the apoplastic and symplastic paths), but also transcellular routes due to the hydraulic barriers in the apoplasts of roots. AQPs play important roles in influencing the rapid flow of water via transcellular pathways and the maintenance of proper *L*_pr_, thereby facilitating root water uptake and transportation [2,3,83,84,85]. AQPs are also helpful for root absorption and the transport of nutrients since some AQPs themselves have the capacity to transfer multiple nutrients like ammonium, urea and boric acid [1,2,86].

Besides PIPs and TIPs, NIPs play a role in root growth. In maize, a mutant *tassel-less1* (*tls1*) has been characterized. The nodal roots of the mutant are clearly shorter than those of normal siblings. In addition, disruption of *TLS1* results in thinning cell walls in the xylem, a reduced Casparian strip and less uniform cell shape (Figure 2a). Further studies revealed that TLS1 is an AtNIP5;1 homolog, and capable of transporting water and boric acid. Loss-of-function mutation of TLS1 causes boron deprivation, reduced dimerization of the pectic polysaccharide rhamnogalacturonan II (RG-II) of cell walls and disorder in meristem function [49] (Table 1).

### 3.3. Hypocotyls and Stems

The growth of hypocotyls and stems is dominantly attributed to cell elongation, which is regulated by AQP-dependent changes in hydraulic conductivity and cell turgor pressure. In *Arabidopsis*, *AtTIP1;1* has been found to be highly expressed in hypocotyls, and its expression pattern is correlated with cell expansion [87]. Similarly, strong expression of *TIP1;1* genes has been found in the growing tissues of shoots in multiple plants like maize, tulip (*Tulipa gesneriana*), cauliflower (*Brassica oleracea*) and oilseed rape [64]. *PsPIP1;1*, *PsPIP2;1* and *PsTIP1;1* are also preferentially expressed in shoots (stems) of pea seedlings [31]. Additionally, in castor bean (*Ricinus communis*), the transcripts and proteins of *RcPIP1;1*, *RcPIP2;1* and *RcTIP1;1* are very abundant in hypocotyl tissues. The abundance of *RcPIP2;1* mRNA is positively correlated with the elongation activity of hypocotyls [88]. Besides, Muto et al. provided evidence that the transcripts of *OsPIP1;1*, *OsPIP1;2*, *OsPIP2;1*, *OsPIP2;6*, *OsTIP1;1*, *OsTIP2;2* and *OsSIP1;1* are enriched in the growing internodes in rice [89]. Both OsTIP1;1 and OsTIP2;2 have water channel activity when expressed in yeast cells. The transcription levels of *SvPIP2;1* are also very high in the elongating stem in *Setaria viridis* [90] (Figure S1). Based on these observations, it is plausible that these PIPs, SIPs and especially TIPs as water transporters function in hypocotyl and stem growth.

Genetic experiments further demonstrated that AQPs are required for the elongation of hypocotyls and stems in plants. It has been reported that overexpression of *AtPIP1b* in tobacco observably increases the height and number of shoot internodes and stem diameter of transgenic plants under favorable growth conditions, but not under drought or salt stresses. Moreover, transpiration rates, photosynthesis rates, photochemical quantum efficiency and stomatal density of the transgenic lines are prominently higher than those of the control plants [50]. Transgenic tobacco plants expressing antisense *BnPIP1* show thicker and shorter stems under normal conditions [51]. Also, the ectopic expression of radish (*Raphanus sativus*) *RasPIP2;1* in *Eucalyptus* trees evidently stimulates the increase in assimilation of CO_2_ and shoot growth, whereas downregulation of *Eucalyptus PIP1* and *PIP2* by introducing *RsPIP1;1* in *Eucalyptus* causes a clear inhibition of CO_2_ assimilation and shoot growth [52]. Similarly, the ectopic expression of rice *OsPIP1;3* in tobacco (*Nicotiana benthamiana*) significantly promotes shoot growth. The transgenic plants show higher photosynthesis rates, *L*_pr_, water use efficiency and biomass compared with the controls [53] (Figure 2c). These PIPs are specifically expressed in the plasma membrane, and might facilitate rapid water movement into or out of the cytoplasm to affect shoot growth through the alterations of leaf photosynthesis capacity, *L*_pr_ or stomatal development.

Pang et al. investigated the role of AtTIP5;1 in *Arabidopsis*, and found that the overexpression of *AtTIP5;1* markedly increases hypocotyl cell elongation under normal or excess boron conditions [54]. Furthermore, AtTIP5;1 acts as an downstream target of GA signaling, positively influencing hypocotyl cell elongation in *Arabidopsis* [55]. In maize, the mutation of *TLS1* (the homolog of *AtNIP5;1*) causes a clear reduction of seedling height, suggesting that TLS1-mediated boron transport and cell wall synthesis are important for shoot elongation [49]. These findings reveal that PIPs, TIPs and NIPs exert favorable effects in hypocotyl or shoot growth. However, transgenic tomato plants overexpressing *Saussurea involucrata SiPIP1;5A* exhibit dwarf phenotypes [56] (Figure 2c) (Table 1), hinting that different AQP isoforms may have distinct roles in affecting shoot elongation in plants.

AQPs also act in the modulation of the relative growth of shoots and roots. For example, Kaldenhoff et al. found that downregulation of *PIP1a* and *PIP1b* in *Arabidopsis* leads to a five-fold decrease in root abundance and a remarkable reduction of shoot/root ratio. Consistently, Aharon et al. demonstrated that the overexpression of *Arabidopsis PIP1b* in tobacco clearly enhances the shoot/root mass ratio due to a nearly 50% increase in shoot weight [50]. Transgenic rice plants overexpressing barley *HvPIP2;1* also show about a 150% increase of shoot/root ratio compared with the control [91]. The reason for this may be that relatively small number of roots in plants with high activities of AQPs is sufficient to supply water for shoot growth. However, overexpressing *OsPIP2;4* in two rice cultivars (Giza178 and IR64) does not cause marked changes in the shoot/root ratio [92], suggesting that the effects of AQPs on shoot/root ratio may depend upon AQP species and plant genotypes.

### 3.4. Leaf Growth

The leaf is the key plant organ where photosynthesis, respiration and transpiration occur. Leaf growth and development greatly depend upon the successful accomplishment of these metabolisms, which involve continuous transport of much water and CO_2_ in leaf cells and are heavily impacted by the expression and activities of AQPs [93,94]. Transcription studies indicate that changes in the expression of *AtTIP1;1* are correlated with cell expansion in *Arabidopsis* leaves [87]. In maize, *ZmPIP1;1*, *ZmPIP1;2*, *ZmPIP1;3*, *ZmPIP2;1* and *ZmPIP2;2* are actively expressed in the elongation zones of leaves. Moreover, the expression of six *ZmPIP1s* (*ZmPIP1;1*, *ZmPIP1;2*, *ZmPIP1;3*, *ZmPIP1;4, ZmPIP1;5*, *ZmPIP1;6*) and six *ZmPIP2s* (*ZmPIP2;1*, *ZmPIP2;2*, *ZmPIP2;3*, *ZmPIP2;4*; *ZmPIP2;5*, *ZmPIP2;6*) is largely dependent on leaf developmental stages. The expression patterns of these genes are correlated with cell water permeability [95]. Besse et al. investigated the relationship between the expression of 23 AQP genes with leaf development in barley, and found that seven genes (*HvPIP1;1*, *HvPIP1;5*, *HvPIP2;2*, *HvPIP2;5*, *HvTIP1;1*, *HvTIP2;3*, and *HvNIP1;1*) are strongly expressed in the elongation zone (Figure S1). These data provide important clues about the involvement of AQPs in leaf growth [96].

Transgenic experiments give further evidence that PIPs and TIPs are necessary for leaf growth. For instance, transgenic tobacco plants expressing antisense *BnPIP1* display deformed leaves. Furthermore, the leaf veins of antisense plants seem to extrude above the leaf surface compared with those of WT plants [51]. Overexpression of *Vicia faba VfPIP1* in *Arabidopsis* causes a clear promotion of leaf growth [41]. Likewise, the overexpression of wheat *TdPIP2;1* in durum wheat cv. Maali enhances leaf growth [43]. Overexpression of *Panax ginseng PgTIP1* in *Arabidopsis* markedly increases the size of leaf mesophyll cells, most likely due to the roles of PgTIP1 in the promotion of water permeability of tonoplasts and cell enlargement [44]. The ectopic expression of citrus *CsTIP2;1*, a highly expressed gene in leaves, in tobacco also pronouncedly promotes leaf growth under normal growth conditions. Additionally, the *CsTIP2;1* transgenic lines have improved water condition and water use efficiency, enhanced mesophyll cell expansion, midrib aquiferous parenchyma abundance, and decreased H_2_O_2_ accumulation in leaves [57] (Figure 2d) (Table 1).

AQPs contribute to leaf growth likely through enlarging the hydraulic conductivity of leaves (*L*_pl_) in growing tissues [3,94]. It has been documented that AQPs account for about 25–50% of leaf *L*_pl_ in sunflower (*Helianthus annuus*), grapevine, diverse deciduous trees and *Arabidopsis* using AQP inhibitors [78,97,98,99]. Transgenic experiments disturbing PIP genes in *Arabidopsis* also revealed that approximately 35% of whole rosette *L*_pl_ and approximately 50% of *L*_pl_ are attributed to AQPs [100]. Similarly, silencing *PsPIP2;1* by VIGS leads to a 29% reduction of *L*_pl_ and the hydraulic conductivity of leaf cells (*L*_plc_) in pea plants [79]. High *L*_pl_ is commonly associated with greater leaf elongation rates [3,94,101]. Instantaneous leaf growth patterns are also considered to be largely driven by hydraulic relations in mangrove *Avicennia marina* [102].

In addition, AQPs play positive roles in leaf growth by affecting CO_2_ transportation, as numerous AQPs have the capacity to transport CO_2_ and are of importance for photosynthesis and leaf growth [9,103]. For instance, NtAQP1 is a CO_2_ permeable PIP in tobacco [104]. Overexpression of *NtAQP1* in tobacco leads to marked increases in membrane permeability for CO_2_ and water, leaf mesophyll CO_2_ conductance, photosynthesis capacity and the promotion of leaf growth of the transgenic lines, whereas the suppression of *NtAQP1* expression causes decreases in the photosynthesis rate [58,105]. Moreover, NtAQP1 can restore the *Arabidopsis* hexokinase 1-mediated growth inhibition of leaves and plants when expressed in tomato via positively influencing the conductance of CO_2_ [106]. In *Arabidopsis*, AtPIP1;2 controls the permeability to CO_2_. Overexpressing *AtPIP1;2* in tobacco noticeably enhances transpiration rates and the photosynthesis efficiency of transgenic plants, whilst mutations in *AtPIP1;2* decrease photosynthesis rates [107,108,109]. Similarly, overexpression of barley *HvPIP2;1* in rice results in enhanced CO_2_ conductance and CO_2_ assimilation in leaves [110], and expressing *Mesembryanthemum crystallinum AQP McMIPB* in tobacco causes enhanced photosynthesis rates, mesophyll conductance to CO_2_ and leaf growth [59]. Transgenic rice lines overexpressing *OsPIP1;2* also display notable increased biomass, yield, net CO_2_ assimilation, photosynthetic capacity and phloem sucrose transport compared with controls [47] (Table 1). Other AQPs that serve as CO_2_ transporters are HvPIP2;1, HvPIP2;2, HvPIP2;3 and HvPIP2;5 in barley [110,111,112], OsPIP1;1 in rice [113], ZmPIP1;5 and ZmPIP1;6 in maize [114], TcAQP1 in *Terfezia claveryi* [115], and PIP1;1 and PIP1;3 in *Populus tremula × alba* [116]. It has been addressed that isoleucine (I-254) at the C-terminal end of the E-loop of *HvPIP2;3* is an essential amino acid residue for CO_2_ permeability in barley [111].

AQPs also play roles in leaf growth by impacting *L*_pr_. It was reported that acid load, treatment with H_2_O_2_ or anoxia results in clear decreases of *L*_pr_, which cause a significant decline in cell turgor in the elongating zone of leaves and xylem water potentials, and the resultant reduction of leaf elongation rates [75]. Similarly, fine-root *L*_pr_ is positively correlated with leaf area and transpiration in vines, and changes in AQP activities affected by drought and ABA impact *L*_pr_ variation and leaf growth [68,117]. Caldeira et al. also demonstrated that rhythmic leaf growth of maize under continuous light is tightly linked to the fluctuations of hydraulic conductance and of the expression of many *PIPs* (*ZmPIP1;1*, *ZmPIP1;2*, *ZmPIP1;3*, *ZmPIP1;5*, *ZmPIP1;6*, *ZmPIP2;1*, *ZmPIP2;2*, *ZmPIP2;3*, *ZmPIP2;4*, *ZmPIP2;5* and *ZmPIP2;6*) in roots [118]. The underlying mechanism may be that changes in *L*_pr_ affected by PIPs or other AQPs cause alterations in the hydraulic conductivity of leaves and whole plants, thus stimulating leaf growth.

Besides, AQPs function in leaf growth via facilitating the transportation of boric acid. TLS1, a NIP in maize, has been shown to transport boric acid. Compared with normal siblings, mutant *tls1* displays shorter and narrower leaves at the middle and lower parts of the plants. Moreover, leaf development is significantly defective in the mutant at the floral transition time [49] (Figure 2d) (Table 1).

## 4. Roles of AQPs in Reproductive Development, Seed Development and Dormancy

Reproduction and seed development are crucial for the survival and population multiplication of angiosperms. They involve flowering (generation of flower buds, formation of various floral organs including sepals, petals, stamens and carpels, flower opening), pollination, pollen germination and pollen tube growth, fertilization, formation of the zygote, and growth and development of embryos, seeds and fruits. During these processes, AQPs are important players in facilitating the transport of water and nutrients across cellular membranes.

### 4.1. Flower Bud Development, Petal Expansion and Flower Opening

Flower buds from fruit trees in temperate and boreal regions typically undergo endodormancy in winter, during which many complex activities involving water consumption are taken place, and AQPs may be required. Transcriptional studies revealed that the expression of the *PpδTIP1* and *PpPIP2* genes is pronouncedly enhanced in peach (*Prunus persica*) flower buds from November to January or February, the stage of bud endodormancy [119]. Yue et al. identified 20 AQP genes in tea (*Camellia sinensis*) plants and found that the expression levels of *CsPIP2;4*, *CsPIP2;5*, *CsTIP1;1*, *CsTIP1;4*, *CsSIP2;1* and *CsXIP* are lower in dormant buds but higher in active buds. These findings signify that the AQP-mediated movement of water and some nutrients likely play roles in bud development.

In maize, the *tls1* mutant of *ZmNIP*, a gene encoding the boric acid transporter, shows abnormal phenotypes during inflorescence development. Compared with normal siblings, the mutant plants have no tassel or defective tassels, and produce no ear or disabled ears. Further studies revealed that the disruption of *TLS1* results in early defects in apical and axillary meristems in the inflorescence [49].

**Figure 3:**
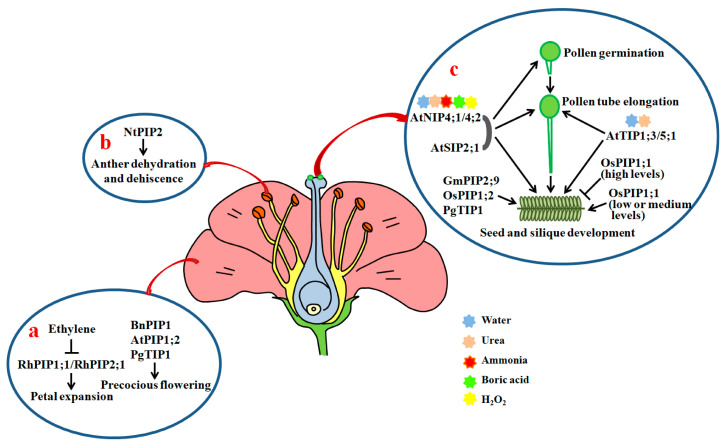
Roles of AQPs in petal expansion, anther dehydration, pollen development, hydration, germination, pollen tube elongation and seed development. (**a**) Roles of AQPs in petal cell expansion and flowering; (**b**) effects of NtPIP2 on anther dehydration and dehiscence; (**c**) AtTIP1;3, AtTIP5;1, AtNIP4;1, AtNIP4;2 and AtSIP2;1 play positive roles in the promotion of pollen hydration, pollen development, pollen tube elongation, and seed and silique development through facilitating the diffusion of water, urea and other small solutes. Arrows show positive regulation and bars show negative regulation.

In rose, *RhPIP2;1* is predominantly expressed in petal epidermal cells, and its expression is strongly linked to petal expansion. Silencing of *RhPIP2;1* by RNAi leads to significant suppression of petal expansion [120]. Further investigations showed that ethylene regulates petal growth by impacting the expression of *RhPIP2;1*. Ethylene is generally regarded as a negative regulator of organ expansion [121]. Treatment with ethylene causes marked inhibition of petal cell expansion and a reduction of water content in rose petals. Moreover, ethylene downregulates the expression of *RhPIP2;1*, and ethylene-treated flowers are similar to those of *RhPIP2;1*-silenced plants in terms of the anatomical characteristics of the petals. Hence, ethylene regulates petal expansion and may rely on the roles of RhPIP2;1 [120]. In addition, RhPIP1;1 can interact with RhPIP2;1 to evidently increase the activity of RhPIP2;1, although RhPIP1;1 alone is incapable of transporting water. Furthermore, suppression of RhPIP1;1 markedly inhibits petal growth [122] (Figure 3a) (Table 2). *RhTIP1;1* has also been shown to be preferentially expressed in rose petals. Its expression is highly correlated with the flowering process, and negatively regulated by ethylene [123].

AQPs may also exert effects on flower opening. In tulip, *TgPIP2;1* and *TgPIP2;2* are abundantly expressed in petals. Petal opening and closing are accompanied by water transport, and are modulated by the reversible phosphorylation of a PIP, most likely TgPIP2;2 [132,133,134]. In tea plants, the transcription of many *CsAQP*s increases at the beginning of flower expansion, and remains at high levels during flower opening [135]. Likewise, AQP genes *DcPIP1;1, DcPIP1;3*, *DcPIP2;1*, *DcPIP2;2*, *DcPIP2;5*, *DcTIP1;4*, *DcTIP2;2*, *DcTIP4;1*, *DcNIP6;1* and *DcSIP1;1* are strongly expressed during all flower opening stages in carnation (*Dianthus caryophyllus*), and high expression levels of *DcPIP1;1* and *DcPIP2;1* are maintained throughout the flower opening process [136,137] (Figure S1). It has been found that expressing antisense *BnPIP1* in tobacco causes a clear delay in blossom time [51]. Similarly, the *Arabidopsis* mutant *atpip1;2* shows delayed flowering time in comparison with the WT [109]. Transgenic *Arabidopsis* plants overexpressing ginseng *PgTIP1* also exhibit the phenotype of precocious flowering as compared with WT plants [44], indicating that PIPs and TIPs have favorable roles in flowering (Figure 3a) (Table 2).

### 4.2. Anther Dehydration, Pollen Development, Hydration and Germination, and Pollen Tube Elongation

In tobacco, *NtPIP1* and *NtPIP2* have shown to be actively expressed in the anther. NtPIP2 protein levels are regulated during anther development. Moreover, downregulation of *NtPIP2* by RNAi results in a clear delay in anther dehydration and dehiscence in comparison with control plants [124,138] (Figure 3b). In rice, *OsPIP1;1* and *OsPIP4;1* are strongly expressed in the anther [35,139] (Figure S1). *PIP1s* are also expressed in the anther in *Brassica* sp. [140]. These findings indicate that PIPs are necessary for the dehydration and dehiscence of anthers.

Recently, Sato and Maeshima found that AtSIP2;1 is localized in the endoplasmic reticulum (ER). A mutation in *AtSIP2;1* results in a significant reduction in the pollen germination rate in comparison with WT. Moreover, the pollen tube lengths of *atsip2;1* are remarkably shorter than those of the WT, and a majority of pollen tubes from *atsip2;1* stop elongating in the mid-region of pistils. Seeds in the bottom region of *atsip2;1* siliques are also scarce, and siliques from *atsip2;1* are evidently shorter than those from WT (Figure 3c). Further, the transcriptional levels of a key ER stress (misfolded protein accumulation in the ER caused by the disorder between the protein folding capacity of the ER and the client protein load) induced gene *Binding protein 3* in pollen in *atsip2;1* are markedly higher than those in WT [125]. This means that AtSIP2;1 exerts positive effects on pollen germination and pollen tube elongation, probably via the alleviation of ER stress in *Arabidopsis*.

In *Arabidopsis*, *AtTIP1;3* and *AtTIP5;1* have been found to be specifically expressed in pollen. The transcripts of *AtTIP1;3* are primarily enriched in vegetative cells, while those of *AtTIP5;1* are abundant in sperm cells or vegetative cells of pollen [126,141]. The elongation of pollen tubes from single mutant *attip1;3* and *attip5;1*, and double mutant *tip1;3/tip5;1* is inhibited compared with the control under nitrogen (N) deprivation conditions. Furthermore, loss-of-function mutations of *AtTIP1;3* and *AtTIP5;1* lead to clear increases in the abnormal rate of barren siliques (Figure 3c). Therefore, both *AtTIP1;3* and *AtTIP5;1* are required for pollen development and pollen tube growth [141]. Since AtTIP1;3 and AtTIP5;1 act as water and urea channels to remobilize N in mature pollen, the two AQPs may function via the transportation of N in *Arabidopsis* [126,142].

AtNIP4;1 and AtNIP4;2 are also pollen-specific water channel proteins, having permeability to ammonia, urea, boric acid, and H_2_O_2_ in addition to water. *AtNIP4;1* is active in mature pollen and pollen tubes with low transcription activity. *AtNIP4;2* is exclusively expressed in pollen tubes, and its expression levels significantly increase during pollen tube growth. Reductions in the expression of *AtNIP4;1* and *AtNIP4;2* by RNAi markedly inhibit pollen germination and pollen tube elongation, and decrease seed fertility. Further studies showed that the water permeable activities of AtNIP4;1 and AtNIP4;2 are regulated by phosphorylation at Ser-267 of the C termini by a pollen-specific calcium-dependent protein kinase 34 [127,143] (Figure 3c). Thus, the kinase-modulated AtNIP4;1 and AtNIP4;2 are important for pollen germination and pollen tube elongation.

In rice, overexpression of *OsPIP1;1* at very high levels markedly decreases fertility, whereas expression of the gene at low or medium levels raises seed yield but does not affect single grain weight [35], suggesting that OsPIP1;1 acts in a seed setting probably via affecting pollen germination in the stigma or pollen tube growth (Table 2). The underlying mechanism remains to be determined.

### 4.3. Fruit Development and Ripening

Fruits are rich in water and various compounds, which are stored within the vacuoles. Fruit growth and development result from cell division, especially cell expansion. Cell expansion largely depends upon AQP-mediated water uptake into the vacuole, which is driven by the high osmotic pressure generated by the compounds. Therefore, AQPs may play important roles in fruit development [144,145]. In pear, TIPs are the most abundant proteins in fruits, and TIP proteins and γ-TIP transcripts are enriched in young fruits. Similarly, peach *γ-TIP* is highly expressed in the early stage and at the end of fruit growth, and changes in the expression of *γ-TIP* appears to be associated with the fruit growth rate [144]. In watermelon (*Citrullus lanatus*), the mRNA of 12 *ClAQP* genes is identified in at least one developmental stage of fruits. Among these, *ClTIP1;2* and *ClTIP1;3* are strongly expressed at four key stages of fruit development [146]. These data provide clues that TIPs are likely more important for fruit growth than other types of AQPs in pear, peach and watermelon.

In tomato, *LePIP1;1*, *LePIP1;4* and *LePIP1;5* are predominantly expressed during fruit development [147]. Both *FaPIP1;1* and *FaPIP2;1* are strongly expressed during fruit ripening in strawberry (*Fragaria × ananassa*) “Camarosa” cultivar. The expression of *FaPIP1;1* is fruit-specific and correlated with fruit ripening [148]. In apple, two genes (*MdPIP1a* and *MdPIP1b*) are highly expressed at the fruit expanding stage [149]. The expression levels of *MdPIP1;3* increase with two peaks, in accordance with the two cell expansion periods during fruit development. Additionally, overexpression of *MdPIP1;3* in tomatoes markedly promotes fruit growth during the expansion stage. Transgenic tomatoes are also significantly bigger and heavier than WT tomatoes [128] (Table 2). These results indicate that PIPs may be the main participators of fruit development in the three plants.

Intriguingly, in tomato cultivar “Micro-Tom”, the transcripts of *SlTIP3;1*, *SlTIP3;2*, *SlPIP1;1*, *SlPIP1;2*, *SlPIP1;7*, *SlNIP2;1*, *SlNIP4;1*, *SlNIP5;1*, *SlNIP6;1*, *SlSIP2;1* and *SlXIP1;1* are rich in fruits, and most of the genes are expressed in a fruit development-specific manner [150]. In grape plants, the transcription of several *AQPs* like *VvTIP1;2*, *VvTIP2;1*, *VvPIP1;2*, *VvPIP1;3*, *VvPIP2;1*, *VvPIP2;3* relies on berry developmental stage. The expression of most of the genes is strong in young berries, and downregulated during berry ripening, while that of *VvTIP1;2*, *VvTIP1;3*, *VvPIP2;3* and *VvPIP2;5* is upregulated at the onset of ripening and later during maturation of the berry. Moreover, the highly expressed AQP genes in young berries are frequently seen in dividing and elongating cells, and in the cells participating in water and solute transport [151,152]. Hu et al. identified 47 AQP genes in banana plants and analyzed the expression patterns of 40 genes at different stages of fruit development and ripening. Twenty-five and thirty genes were found to be expressed in the “FJ” and “BX” varieties, respectively, during all stages of fruit development. *MaPIP2;10*, *MaPIP1;6*, *MaPIP2;7*, and *MaSIP1;1* in “FJ”, and *MaTIP4;1* and *MaPIP2;5* in “BX” show high expression at all tested stages [153] (Supplementary Figure S1). Likewise, 12 *CsPIP* genes revealed to be differentially expressed during fruit development in cucumber plants, and *CsTIP1;1*, *CsTIP2;1* and *CsPIP1;3* are expressed in dynamic and fruit-specific patterns [154,155]. It appears that TIPs and PIPs, as well as NIPs, SIPs and XIPs, are modulators of fruit development in these plants.

### 4.4. Seed Development and Dormancy

Generally, seed development can be divided into three stages: the early cell division and embryogenesis after the double fertilization, seed maturation (including cell expansion, differentiation and the accumulation of storage compounds in the embryo), and late maturation of seeds including the acquisition of desiccation tolerance [156,157]. During the first two stages, substantial changes in the water and solute contents occur in seeds, and high amounts of water and nutrients are required for cell division, elongation and differentiation. As essential facilitators of water and solutes, AQPs may be involved in the transportation of these substances required for seed development. At the late stages of maturation, especially for orthodox seeds, AQPs serve roles in rapid water efflux, leading to seed dehydration and the accumulation of large amounts of dry mass [25,39].

Transcription analyses revealed that many AQP genes are actively expressed in diverse seed tissues and at different stages of seed development in plants. In *Arabidopsis*, transcripts of *AtPIP1;2*, *AtPIP1;3*, *AtPIP1;4*, *AtPIP1;5*, *AtPIP2;1*, *AtPIP2;2/2;3*, *AtPIP2;5*, *AtPIP2;7/AtPIP2;8*, *AtTIP1;1*, *AtTIP2;2*, *AtTIP3;1*, *AtSIP1;1*, *AtSIP1;2* and *AtSIP2;1* are abundant in one or more tissues, and at certain stages of seed development [25]. Also, a number of genes are strongly expressed in the early periods of seed development, like *ScPIP2s* in the ovules of *Solanum chacoense*; *PtNIP1;1* in the suspensors of *Pinus taeda*; *PsPIP1;1*, *PsPIP2;1*, *PsTIP1;1* and *PsNIP1;1* in the expanding cotyledons and seed coats of pea [31]; *LePIP1;1*, *LePIP1;2*, *LePIP1;4*, *LePIP1;5* and *LePIP2;1* in tomato [147]; *PvPIP1;1*, *PvPIP2;2* and *PvPIP2;3* in seed coats of French bean [22,158]; and *OsPIP1;1*, *OsPIP1;2*, *OsPIP2;1*, *OsPIP2;2*, *OsPIP2;6*, *OsTIP2;2*, *OsTIP4;2*, and *OsNIP1;1* in rice [159], pointing to the potential roles of these AQPs in seed development (Figure S1).

In soybean (*Glycine max*), *GmPIP2;9* is highly expressed in developing pods and the seed hilum, where assimilation and water transport occur. GmPIP2;9 has high water channel activity. Moreover, overexpression of *GmPIP2;9* causes pronounced increases in the pod number, seed number, and seed weight per plant, indicating that GmPIP2;9 may facilitate water flow in pod walls from the seed coat, and play a positive role in developing seeds, seed setting and filling [129]. In rice, overexpression of *OsPIP1;2* markedly enhances the number of spikelets per panicle and yield, reflecting the roles of the AQP in seed development. The major mechanism for this may be that OsPIP1;2 favors mesophyll CO_2_ conductance, further causing the enhancements of net CO_2_ assimilation rate, photosynthetic capacity and phloem sucrose transport, but not due to OsPIP1;2 facilitation of water transport in leaves [47]. Besides, overexpression of *Panax ginseng PgTIP1* in *Arabidopsis* promotes the development of seeds [44] (Table 2).

AQPs are also of importance for seed maturation. During the maturation of orthodox seeds, up to 90% of water is lost. Concomitantly, LVs convert into numerous PSVs, and large quantities of protein reserves are accumulated in the PSVs. Accompanied by the formation of PSVs, compositions of TIP3s significantly increase, while those of TIP1s noticeably decrease in seeds. Moreover, the expression of *TIP3s* and *TIP1s* genes is remarkably altered [28,160]. In *Arabidopsis*, the transcripts and/or proteins of *AtTIP3;1* and *AtTIP3;2* are found to be abundant during seed maturation [26,28]. Moreover, disruptions of both *AtTIP3;1* and *AtTIP3;2* genes clearly reduce seed longevity and increase the accumulation of H_2_O_2_ in seeds. The promoters of *AtTIP3;1* and *AtTIP3;2* can be bound and activated by the transcription factor ABA insensitive 3, a master regulator of seed maturation, in the presence of ABA [130]. Thus, AtTIP3;1 and AtTIP3;2 act as positive modulators of seed maturation and seed longevity by transporting water and H_2_O_2_ under the control of ABA.

In rice, the transcripts and protein contents of *OsPIP2;1*, *OsTIP2;2* and *OsTIP3;1* are very abundant in the mid-grain filling stage of seeds. OsPIP2;1 mainly accumulates in the starchy endosperm, nucellar projection, nucellar epidermis, and dorsal vascular bundles, while OsTIP3;1 is enriched in the aleurone layer and starchy endosperm during rice grain filling [159]. Besides, barley *HvTIP3;1* has been shown to be specifically expressed after the middle ripening stage of seed development, with its expression reaching a peak during seed desiccation. HvTIP3;1 proteins accumulate in aleurone cells and in the outer layers of developing seeds, and have water permeability when co-expressed with HvTIP1;2 in *Xenopus oocytes* [161]. AhTIP3;1 is also implicated in the formation of small vacuoles in the maturing seeds of horse chestnut [61]. Therefore, PIP2s, TIP2s and TIP3s may function in seed maturation by transporting water and solutes.

AQPs also play roles in seed dormancy. Footitt et al. provide genetic evidence that AtTIP3;1, AtTIP3;2 and AtTIP4;1 are involved in the modulation of seed dormancy. All three TIPs have inhibitory roles in primary dormancy induction. AtTIP3;1 and AtTIP3;2 act antagonistically in the induction of secondary dormancy. AtTIP3;2 and AtTIP4;1 exert repressed effects, but AtTIP3;1 has promoted effects on the secondary dormancy induction during dormancy cycling in *Arabidopsis*. Gene and protein expression of *AtTIP3;1* and *AtTIP3;2* is also changed during seasonal dormancy cycling. The expression levels increase when seed dormancy levels enhance, and vice versa [39] (Table 2).

### 4.5. Fiber Elongation

Cotton fibers are unicellular trichomes that originate from the ovule epidermis. Their formation is due to the rapid growth of fiber cells. AQPs function in fiber elongation. It is reported that the genes *GhγTIP1*, *GhPIP1;2*, *GhPIP2;3*, *GhPIP2;4*, *GhPIP2;5* and *GhPIP2;6* are preferentially expressed in fibers in *Gossypium hirsutum*, and the transcripts of the latter four *GhPIP2s* reach peak values in the stage of fiber cell rapid elongation. Moreover, the overexpression of *GhPIP2;3*, *GhPIP2;4* or *GhPIP2;6* in yeast markedly promotes longitudinal growth of the host cells. Knockdown of *GhPIP2s* in cotton by RNAi clearly inhibits fiber elongation. In addition, *GhPIP2;3*, *GhPIP2;4*, *GhPIP2;5* and *GhPIP2;6* are able to form heterotetramers, and their water channel activities increase accordingly [162]. Transcriptome analyses also revealed that the expression of seven *PIPs*, four *TIPs* and two *NIPs* in cotton short fiber mutants *Li1* and *Li2* remarkably decreases in developing fibers as compared with WT plants. Additionally, the osmotic pressure of fiber cells in the mutants is markedly reduced during the rapid cell elongation of fibers [163] (Table 2). These findings highlight the possible roles of the PIPs, TIPs and NIPs in the osmoregulation of fiber growth in cotton.

The roles of AQPs in fiber cell elongation are also confirmed in Milkweed (*Calotropis procera*), a kind of plant generating long seed trichomes. In the plant, the *CpPIP2* gene is strongly expressed in trichomes, and peaks in its expression when trichome cells elongate at the highest rate. Furthermore, overexpression of *CpPIP2* in tobacco prominently increases the number of trichomes on leaves and stems [131] (Table 2).

## 5. AQPs Serve Roles in Plant Growth and Development through Transferring ROS across Membranes

Reactive oxygen species (ROS) such as the superoxide radical and H_2_O_2_ are toxic byproducts of cellular metabolism in plants. They also act as signal molecules to regulate stomatal movement, programmed cell death, hormone signal transduction, plant responses to diverse environmental stimuli, as well as growth and development, including seed development and germination, primary root elongation, lateral root branching, root hair formation, shoot growth and flower development [164,165,166,167,168,169,170]. Several lines of evidence suggest that plant AQPs can conduct H_2_O_2_ across the biological membrane. For instance, *Arabidopsis* AtTIP1;1, AtTIP1;2, AtTIP2;3, AtPIP2;1, AtPIP2;2, AtPIP2;4, AtPIP2;5, AtPIP2;7 and AtNIP1;2 are permeable to H_2_O_2_ in yeast cells [171,172,173,174]. It has also been found that rice OsNIP3;2, OsNIP3;3 and OsTIP2;2, barley HvPIP2;5, HvTIP2;2, HvTIP2;3 and HvTIP5;1, maize ZmPIP2;5, tulip TgTIP1;1 and TgTIP1;2, tobacco NtXIP1;1, tomato SlXIP1;1 and potato StXIP1;1 transport H_2_O_2_ in yeast [175,176,177,178,179]. Moreover, AtPIP2;1, AtPIP1;4 and other AtPIP2s facilitate H_2_O_2_ transportation in *Arabidopsis* [180,181,182].

AQPs may play essential roles in the detoxification and signaling of ROS through facilitating intracellular translocation of H_2_O_2_ during growth and development. In plants, H_2_O_2_ is mainly generated in the apoplast, plasma membrane, chloroplast, mitochondria, peroxisome and ER [183]. AtPIP1;4, has been shown to transfer apoplast H_2_O_2_ induced by bacterial pathogen and pathogen-associated molecular patterns (PAMPs) to the cytoplasm to activate systemic acquired resistance and immune responses in *Arabidopsis* [181]. Likewise, AtPIP2;1 mediates the transportation of H_2_O_2_ from the apoplast to the cytoplasm of guard cells during ABA- and PAMP flg22-triggered stomatal closure [182]. In addition, AtPIP2;1 favors the movement of both H_2_O_2_ and water during stomatal closure [182,184]. AtPIP2;4 and spinach SoPIP2;1 also transport both H_2_O and H_2_O_2_ [185], implying that many water permeable AQPs are H_2_O_2_ transporters in plants. Indeed, five out of 13 Arabidopsis AtPIPs can conduct H_2_O_2_ in yeast [173]. It seems that some AQPs are localized in the chloroplast envelope and aid the transport of H_2_O_2_ from the inside to the outside of the chloroplast, since the AQP inhibitor acetazolamide significantly suppresses H_2_O_2_ release from isolated chloroplasts under high light [186]. H_2_O_2_ derived from the chloroplast has been found to affect the level of nuclear H_2_O_2_ and gene expression [187]. Accordingly, changes in the activities of chloroplast envelope AQPs likely impact light signaling and plant growth. Some AQPs like AtTIP5;1 have been documented or predicted to be localized to the mitochondria in plants [126,188], yet it is unclear whether these AQPs transfer H_2_O_2_ across the mitochondrial membrane. To date, no AQP has been detected in plant peroxisomes. However, evidence reveals that porin-like channels or proteins exist in the peroxisomal membrane in castor bean, sunflower and cotton plants. These porins transfer small metabolites like succinate, malate and aspartate through the membrane [189,190]. They probably facilitate H_2_O_2_ transport across peroxisomal membrane under specific conditions. Several AQPs like Arabidopsis SIP1;1, SIP1;2 and SIP2;1 are localized to the ER, and the first two AQPs, but not the third, show permeability to water. SIP2;1 acts in pollen germination and pollen tube growth possibly through the alleviation of ER stress, and is an ortholog of mammalian AQP11 [125]. Recently, AQP11 has been demonstrated to transport H_2_O_2_ out of the ER [191]. It is possible that SIP2;1 is a H_2_O_2_ transporter during pollen development. Whether SIP2;1 is permeable to H_2_O_2_ deserves further investigation.

In the presence of ABA, the H_2_O_2_ transport activity of AtPIP2;1 is activated by the brassinosteroid insensitive 1-associated receptor kinase 1 and/or open stomata 1/Snf1-related protein kinase 2.6-mediated phosphorylation of Ser121 [182,184]. The activities and expression of AQPs are also modulated by H_2_O_2_ levels. H_2_O_2_ treatment clearly inhibits the water transport capacity of AQPs in maize [192]. H_2_O_2_ application also changes AQP expression in *Arabidopsis* [173], indicating that a feedback regulation of AQP-facilitated H_2_O_2_ diffusion may exist. Besides, alterations in the H_2_O_2_ level can influence the subcellular localization of an AQP. H_2_O_2_ treatment results in the redistribution of AtPIP2;1 from the plasma membrane to the endomembane [193]. Therefore, the distribution and permeability of AQPs and H_2_O_2_ levels are dynamic and mutually affected, and AQPs may play roles in growth and development as ROS signaling transducers and modulators of ROS homoeostasis in plants.

## 6. Concluding Remarks and Perspectives

AQPs are widely distributed in the plasma membrane and other organellar membranes, and play versatile roles in nearly every aspect of plant growth and development by affecting the water homeostasis of cells and whole plants, leaf photosynthesis capacity and cell wall formation, among others. Accumulating evidence suggests that TIPs are essential for cell expansion since they predominantly contribute to the formation of large central vacuoles and the buildup of cell turgor pressure, the driving force for cell extension. PIPs seem to function in cell division in plants [2,36,63]. By contrast, NIPs are required for cell division and cell elongation primarily via the transportation of nutrients like boric acid. Boron participates in cell wall expansion and is crucial for cell formation and cellular functions. Yet, information on the involvement of SIPs and XIPs in growth and development is relatively scarce.

In the past few decades, much progress has been achieved in understanding the functions and regulatory mechanisms of various AQPs. Enormous transcriptional evidence and a great number of forward and reverse genetic data concerning the roles of AQPs in plant growth and development have been accumulated. However, it is still very hard to define the precise contributions of individual AQPs to growth and development in plants because water is ubiquitous and transported along not only the AQP-involved transcellular pathway, but also xylem vessels, and the apoplastic and symplastic routes. Moreover, a plant commonly has a number of AQP members which operate redundantly and mutually affect each other [2,161]. AQPs can also interplay with other proteins or exogenous AQPs (ectopic expression of *AQPs*), and the expression and activities of numerous AQPs are regulated by many mechanisms [3,194]. Consequently, the effects of one AQP on growth and development almost integrate with those of other AQP homologs or proteins. Therefore, researchers need to develop new technologies and methods to unveil the specific roles and underlying mechanisms of individual AQPs in plant growth and development.

It is well known that plant growth and development are ultimately controlled by various phytohormones. AQPs commonly act downstream of hormones signaling and are downstream targets of some hormones such as auxin, GA, ABA and ethylene in root formation, seed germination or petal expansion [38,45,195]. However, the detailed mechanisms for most of the AQPs are still not clear. Moreover, it remains to explore whether and how other hormones like brassinostroid, cytokinin and salicylic acid modulate plant growth and development through affecting the expression and activities of AQPs.

As described above, AQPs play important roles in plant growth and development under diverse environmental stimuli. The effects of RhPIP2;1 in the balance between growth and survival is emerging [196]. Yet, our knowledge about the role of AQPs in the coordination of different growth and stress signals through the exchange of water and solutes across biological membranes, and the underlying mechanisms, is largely lacking.

At present, AQPs have been considered to be important candidate genes for engineering to improve crop stress tolerance and yields [42,197]. Therefore, much effort should be made to better understand the complex regulatory mechanisms of AQPs to optimize the efficient utilization of water and nutrients for healthy plant development, especially under stress conditions. We believe that the specific functional mechanisms of AQPs will be elucidated with the development of various omics technologies and other new technologies like CRISPR–Cas9 in the future.

## Figures and Tables

**Table 1:** The roles of AQPs in the growth of vegetative organs.

Organs	Transgenic Plants or Mutants	Receptors or Studied Plants	Phenotypes	AQP Functions	References
Seeds	K: *OsPIP1;3*	*O. sativa*	Reduced seed germination	Water transport	[29]
	O: *OsPIP1;1*	*O. sativa*	Enhanced seed germination rate	Water permeability	[35]
Roots	K: *AtPIP1b*	*A. thaliana*	Abundant roots	Water transport	[40]
	O: *VfPIP1*	*A. thaliana*	Increased growth of primary and LRs	-	[41]
	O: *ScPIP1*	*A. thaliana*	Promoted primary root growth	-	[42]
	O: *TdPIP2;1*	*T. turgidum*	Increased root length	-	[43]
	O: *PgTIP1*	*A. thaliana*	Increased primary root length	Water transport	[44]
	O: *AtPIP2;1*	*A. thaliana*	Delayed LR emergence	Water transport	[45]
	M: *attip2;1*	*A. thaliana*	Delayed LR emergence and delayed LRP development	Water transport	[45,46]
	M: *attip1;1*	*A. thaliana*	Increased root growth at early seedling stage and delayed LRP development	-	[46]
	M: *attip1;2*	*A. thaliana*	Delayed LRP development	-	[46]
	M: *attip1;2/2;1*	*A. thaliana*	Delayed LRP development	-	[46]
	M: *attip1;1/1;2/2;1*	*A. thaliana*	Reduced root growth, fewer LRs and decreased LR emergence	-	[46]
	O: *OsPIP1;2*	*O. sativa*	Increased root length	CO_2_ transport	[47]
	R: *OsPIP2;1*	*O. sativa*	Decreased root length, surface area, root volume and root tip number	Water transport	[48]
	M: *tls1*	*Z. mays*	Decreased length of nodal roots	Boric acid transport	[49]
Shoots/Stems/Hypocotyls	O: *AtPIP1b*	*N. tabacum*	Increased length and number of shoot internodes and stem diameter	-	[50]
	R: *BnPIP1*	*N. tabacum*	Thicker and shorter stems	-	[51]
	*O: RsPIP2;1*	*E. grandis and ×E. urophylla*	Increased shoot growth	-	[52]
	*O: RsPIP1;1* (Downregulation of *EcPIP1* and *EcPIP2*)	*E. grandis× E. urophylla*	Decreased shoot growth	Suppression of the endogenous expression of *EcPIP1* and *EcPIP2*	[52]
	O: *OsPIP1;3*	*N. benthamiana*	Increased shoot growth	Water transport	[53]
	O: *AtTIP5;1*	*A. thaliana*	Increased hypocotyl cell elongation	-	[54,55]
	M: *tls1*	*Z. mays*	Decreased shoot elongation	Boric acid transport	[49]
	O: *SiPIP1;5A*	*S. lycopersicum*	Decreased shoot elongation	-	[56]
Leaves	R: *BnPIP1*	*N. tabacum*	Deformed leaves and leaf veins	-	[51]
	O: *VfPIP1*	*A. thaliana*	Larger leaves	-	[41]
	O: *TdPIP2;1*	*T. turgidum*	Enhanced leaf growth	-	[43]
	O: *PgTIP1*	*A. thaliana*	Increased size of leaf mesophyll cells	Water transport	[44]
	O: *CsTIP2;1*	*N. tabacum*	Increased leaf growth	-	[57]
	O: *NtAQP1*	*A. thaliana*	Increased leaf area and number	CO_2_ permeability	[58]
	O: *McMIPB*	*N. tabacum*	Enhanced leaf growth	CO_2_ diffusion	[59]
	M: *tls1*	*Z. mays*	Decreased leaf size and number	Boric acid transport	[49]

K: knockdown plants; LRs: lateral roots; LRP: lateral root primordium; M: mutants; O: overexpressors; R: RNAi plants; *tls1*: *maize tassel-less1*; -: no mentioned transport activity.

**Table 2:** The roles of AQPs during reproductive organ development.

Organs	Transgenic Plants or Mutants	Receptors or Studied Plants	Phenotypes	Role(s) of the AQPs	References
Flower buds	M: *tls1*	*Z. mays*	Defects in apical and axillary meristems of inflorescence	Boric acid transport	[49]
Petals	R: *RhPIP2;1*	*R. hybrida*	Decreased petal expansion	Transport of water	[120]
	R: *RhPIP1;1*	*R. hybrida*	Inhibited petal expansion	Interaction with RhPIP2;1	[122]
Flowering	R: *BnPIP1*	*N. tabacum*	Delayed flowering time	-	[51]
	M: *atpip1;2*	*A. thaliana*	Delayed flowering time	CO_2_ diffusion	[109]
	O: *PgTIP1*	*A. thaliana*	Promoted precocious flowering	Water transport	[44]
Anthers	R: *NtPIP2*	*N. tabacum*	Delayed anther dehydration and dehiscence	Water transport	[124]
Pollen	M: *atsip2;1*	*A. thaliana*	Defect in pollen germination and pollen tube elongation	-	[125]
	M: *attip1;3* M: *attip5;1* M: *attip1;3/5;1*	*A. thaliana*	Inhibited pollen tube elongation	AtTIP5;1: nitrogen transport	[126]
	R: *AtNIP4;1* R: *AtNIP4;2*	*A. thaliana*	Reduced pollen germination and pollen tube length	AtNIP4;1: transport of water, ammonia, urea, boric acid, H_2_O_2_ and glycerol AtNIP4;2: transport of water and glycerol	[127]
Seeds/Fruits	R: *AtNIP4;1* R: *AtNIP4;2*	*A. thaliana*	Fewer seeds per silique	AtNIP4;1: transport of water, ammonia, urea, boric acid, H_2_O_2_ and glycerol AtNIP4;2: transport of water and glycerol	[127]
	O (high levels): *OsPIP1;1*	*O. sativa*	Decreased seed fertility	A putative water transporter	[35]
	O (low or medium levels): *OsPIP1;1*	*O. sativa*	Increased seed yields	A putative water transporter	[35]
	O: *MdPIP1;3*	*S. lycopersicum*	Promoted expanding growth of fruits	Water transport	[128]
	O: *GmPIP2;9*	*G. max*	Increased pod number, seed number and seed weight per plant	Water transport	[129]
	O: *OsPIP1;2*	*O. sativa*	Enhanced number of spikelets per panicle and seed yield	A putative CO_2_ transporter	[47]
	O: *PgTIP1*	*A. thaliana*	Promoted seed development	Water transport	[44]
	M: *attip3;1* M: *attip3;2*	*A. thaliana*	Decreased seed longevity	-	[130]
	M: *attip3;1* M: *attip4;1*	*A. thaliana*	Inhibited primary and secondary seed dormancy	-	[39]
	M: *attip3;2*	*A. thaliana*	Inhibited primary seed dormancy and promoted secondary seed dormancy	-	[39]
	O: *CpPIP2*	*N. tabaccum*	Increased trichome cell elongation	-	[131]

K: knockdown plants; M: mutants; O: overexpressors; R: RNAi plants; *tls1*: *maize tassel-less1*; -: no mentioned transport activity.

## Supplementary Materials

Supplementary materials can be found at https://www.mdpi.com/1422-0067/21/24/9485/s1.

## Abbreviations

ABA	Abscisic acid
AEF or AEFXXT	Ala-Glu-Phe
AQPs	Aquaporins
ar/R	Aromatic/arginine
CO_2_	Carbon dioxide
ER	Endoplasmic reticulum
GAs	Gibberellins
GLAs	Aquaglyceroporins
GLPs/GlpFs	Glycerol-facilitators
g_s_	Stomatal conductance
H_2_O_2_	Hydrogen peroxide
*L*_pl_	Hydraulic conductivity of leaves
*L*_pr_	Hydraulic conductivity of roots
*L*_prc_	Hydraulic conductivity of root cells
LR	Lateral root
LRPs	Lateral root primordia
LVs	Lytic vacuoles
MIPs	Intrinsic superfamily proteins
N	Nitrogen
NIPs	Nodulin 26-like intrinsic proteins
NPA	Asp-Pro-Ala
PAMP	Pathogen-associated molecular pattern
PEG	Polyethylene glycol
PIPs	Plasma membrane intrinsic proteins
ROS	Reactive oxygen species
Si	Silicon
SIPs	Small basic intrinsic proteins
TIPs	Tonoplast intrinsic proteins
WT	Wild type
XIPs	Uncategorized intrinsic proteins
