# Supplementary material for: Versatile Roles of Aquaporins in Plant Growth and Development
Source: Int J Mol Sci. 2020 Dec 13;21(24):9485. doi: 10.3390/ijms21249485 (PMC7763978; doi:10.3390/ijms21249485)
Supplement: Supplementary file 1 [file ijms-21-09485-s001.pdf]

Figure S1 Expression of AQPs during plant growth and development

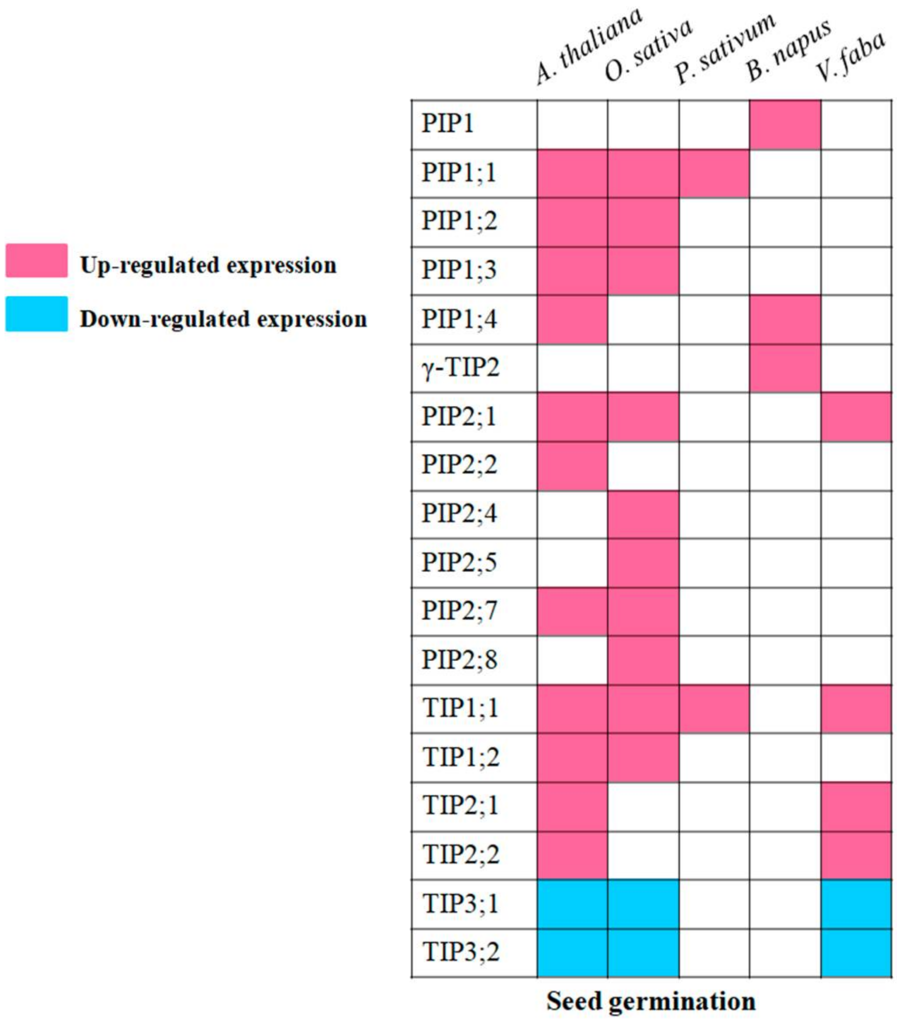

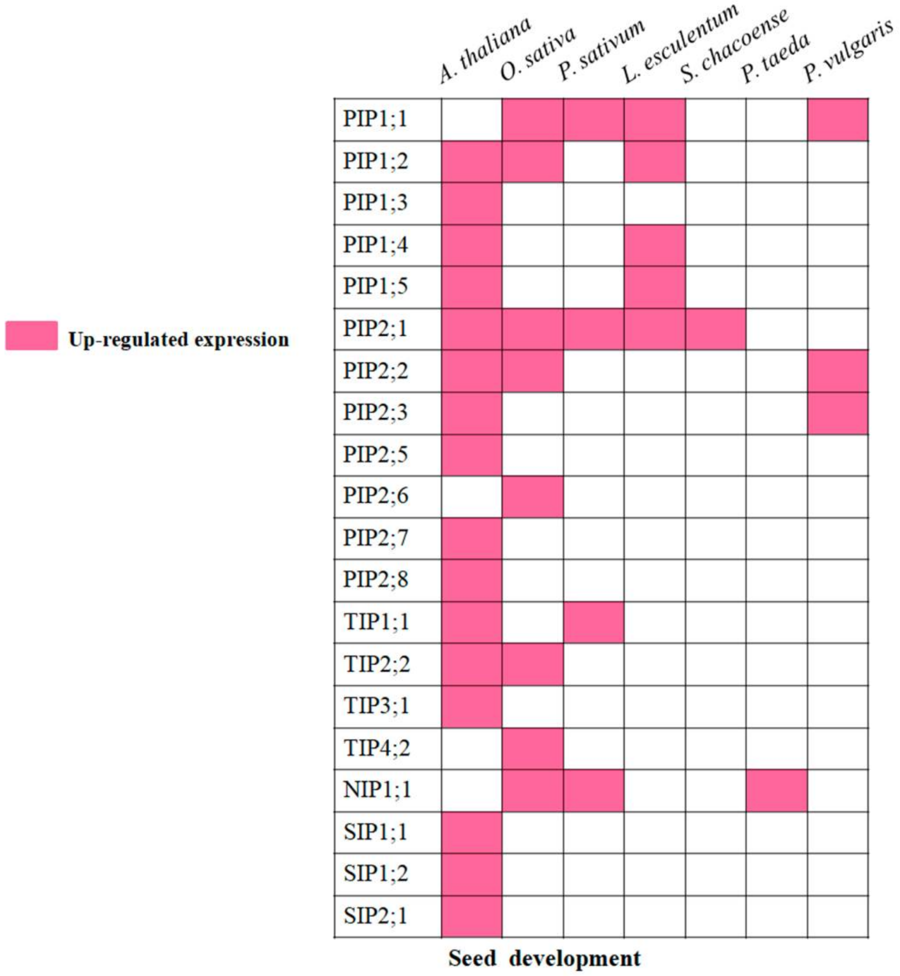

Up-regulated expression
A. thaliana
O. sativa
P. sativum
L. esculentum
S. chacoense
P. taeda
P. vulgaris
PIP1;1
PIP1;2
PIP1;3
PIP1;4
PIP1;5
PIP2;1
PIP2;2
PIP2;3
PIP2;5
PIP2;6
PIP2;7
PIP2;8
TIP1;1
TIP2;2
TIP3;1
TIP4;2
NIP1;1
SIP1;1
SIP1;2
SIP2;1
Seed development

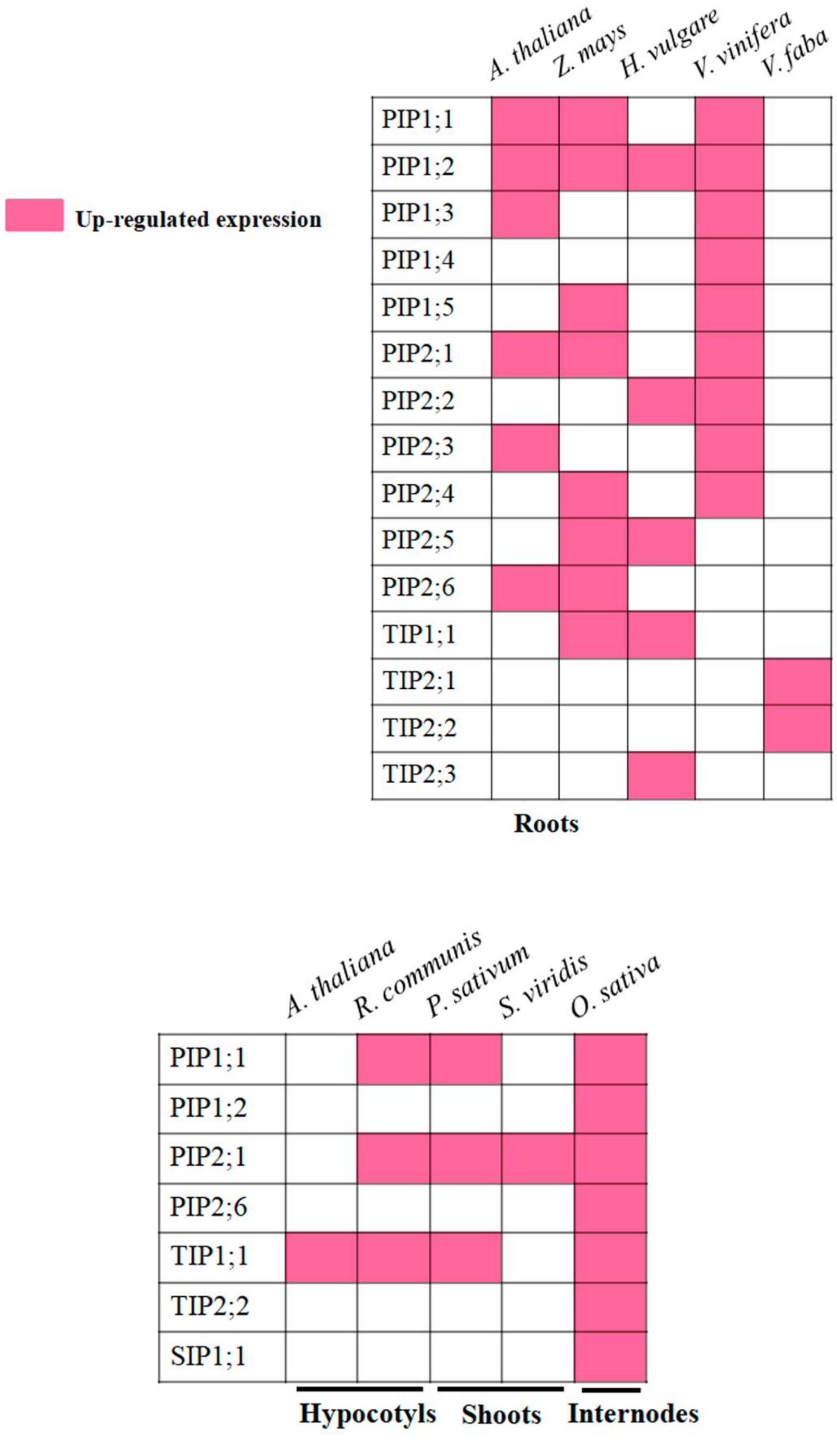

Up-regulated expression
A. thaliana
Z. mays
H. vulgare
V. vinifera
V. faba
PIP1;1
PIP1;2
PIP1;3
PIP1;4
PIP1;5
PIP2;1
PIP2;2
PIP2;3
PIP2;4
PIP2;5
PIP2;6
TIP1;1
TIP2;1
TIP2;2
TIP2;3
Roots
A. thaliana
R. communis
P. sativum
S. viridis
O. sativa
PIP1;1
PIP1;2
PIP2;1
PIP2;6
TIP1;1
TIP2;2
SIP1;1
Hypocotyls
Shoots
Internodes

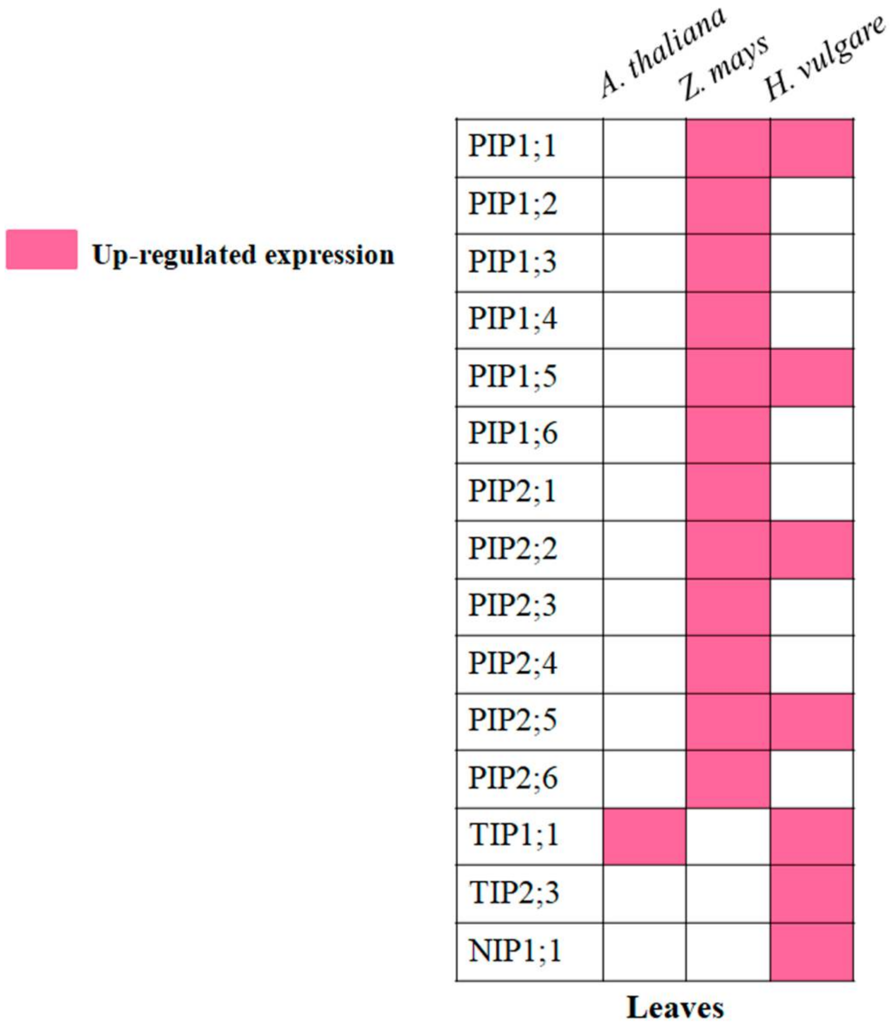

Up-regulated expression
A. thaliana
Z. mays
H. vulgare
PIP1;1
PIP1;2
PIP1;3
PIP1;4
PIP1;5
PIP1;6
PIP2;1
PIP2;2
PIP2;3
PIP2;4
PIP2;5
PIP2;6
TIP1;1
TIP2;3
NIP1;1
Leaves

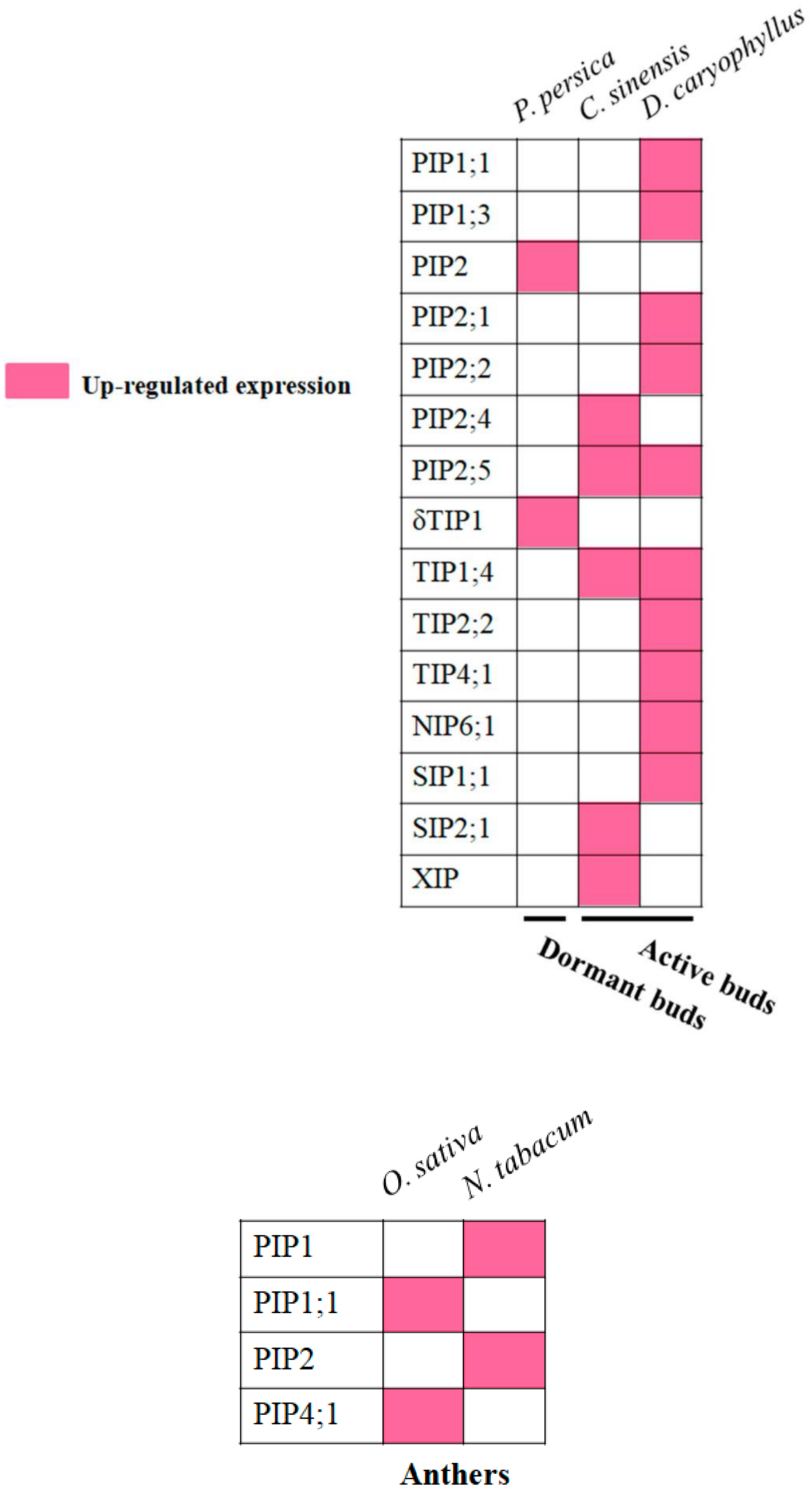
Up-regulated expression
P. persica
C. sinensis
D. caryophyllus
PIP1;1
PIP1;3
PIP2
PIP2;1
PIP2;2
PIP2;4
PIP2;5
δTIP1
TIP1;4
TIP2;2
TIP4;1
NIP6;1
SIP1;1
SIP2;1
XIP
Dormant buds
Active buds
O. sativa
N. tabacum
PIP1
PIP1;1
PIP2
PIP4;1
Anthers

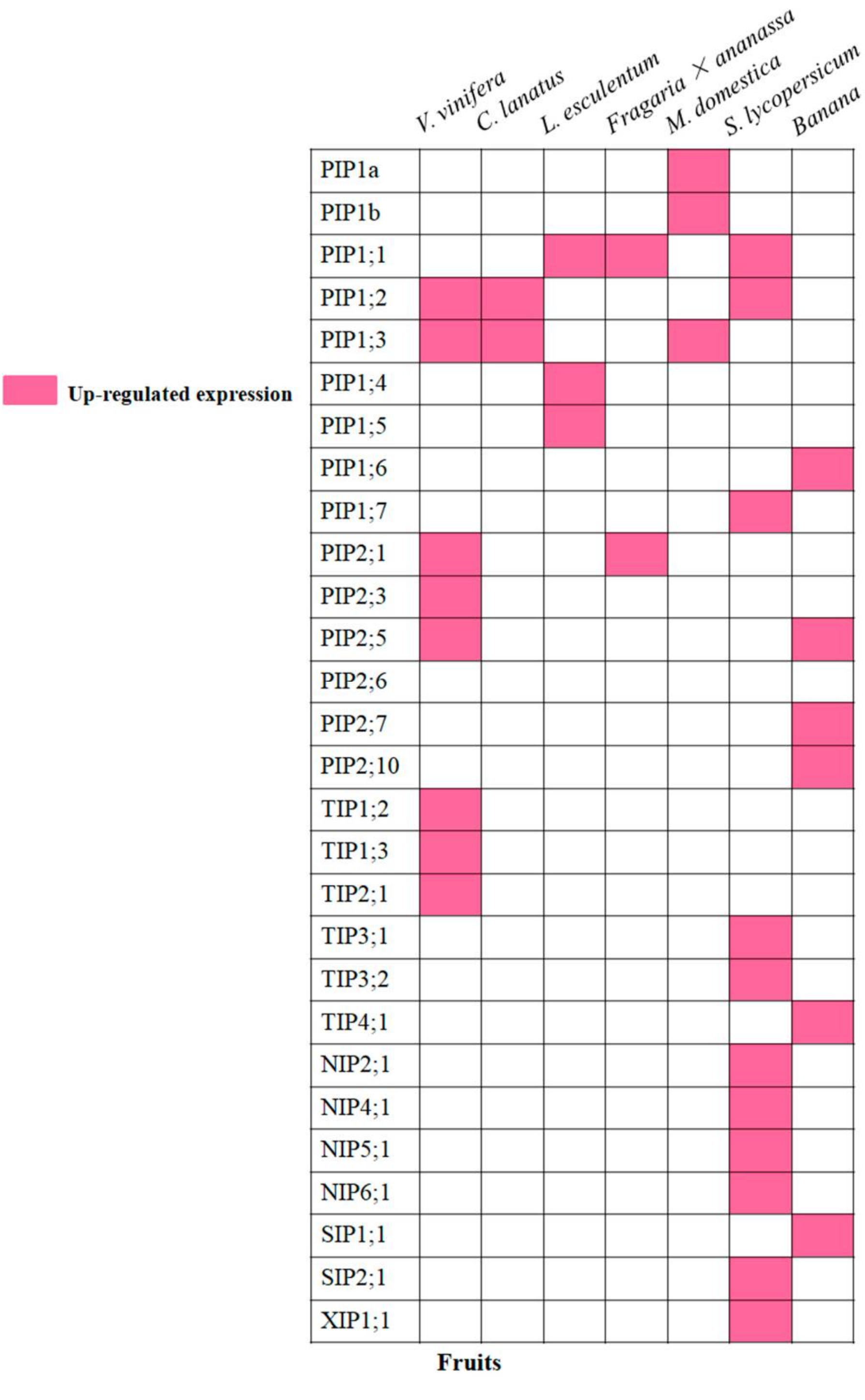
V. vinifera
C. lanatus
L. esculentum
Fragaria × ananassa
M. domestica
S. lycopersicum
Banana
PIP1a
PIP1b
PIP1;1
PIP1;2
PIP1;3
PIP1;4
PIP1;5
PIP1;6
PIP1;7
PIP2;1
PIP2;3
PIP2;5
PIP2;6
PIP2;7
PIP2;10
TIP1;2
TIP1;3
TIP2;1
TIP3;1
TIP3;2
TIP4;1
NIP2;1
NIP4;1
NIP5;1
NIP6;1
SIP1;1
SIP2;1
XIP1;1
Up-regulated expression
Fruits
